# The interplay between innate lymphoid cells and microbiota

**DOI:** 10.1128/mbio.00399-23

**Published:** 2023-06-15

**Authors:** Rui Wang, Wenwen Cui, Huan Yang

**Affiliations:** 1 Xuzhou Key Laboratory of Laboratory Diagnostics, School of Medical Technology, Xuzhou Medical University, Xuzhou, China; Ohio State University, Columbus, Ohio, USA; Ohio State University, Columbus, Ohio, USA

**Keywords:** ILC, microbiota, adaptive immune, DC, metabolite

## Abstract

Innate lymphoid cells (ILCs) are mainly resident in mucosal tissues such as gastrointestinal tract and respiratory tract, so they are closely linked to the microbiota. ILCs can protect commensals to maintain homeostasis and increase resistance to pathogens. Moreover, ILCs also play an early role in defense against a variety of pathogenic microorganisms including pathogenic bacteria, viruses, fungi and parasites, before the intervention of adaptive immune system. Due to the lack of adaptive antigen receptors expressed on T cells and B cells, ILCs need to use other means to sense the signals of microbiota and play a role in corresponding regulation. In this review, we focus on and summarize three major mechanisms used in the interaction between ILCs and microbiota: the mediation of accessory cells represented by dendritic cells; the metabolic pathways of microbiota or diet; the participation of adaptive immune cells.

## INTRODUCTION

It is estimated that the microbiota colonizing in the human gut, including bacteria, fungi, and viruses, have about 100 trillion cells, with far more numbers and gene expressions than the host cells (1). At steady state, members of the microbiota in human body are often referred to as commensals. Furthermore, colonization of commensals competitively suppresses the invasion of pathogens and improves immunity to pathogens through training and regulating immune system (2). In addition, commensals can also help the host digest food and produce essential nutrients for human body (3). However, in consideration of the huge number and species, the commensals pose a persistent invasion risk to human body. For instance, many opportunistic pathogens exhibiting pathogenicity after translocation or local environmental change (4). No matter commensals or pathogens, the immune system is required to regulate the microbiota-host relationship.

Immune system includes innate components and adaptive components (5). The innate components mainly consist of mucosal barriers and epithelial tight junctions that provide mechanical immunity by reducing contact with microbiota; and innate lymphoid cells (ILCs) and monocytes that provide effector immunity by producing cytokines (6). Among them, ILCs have many subsets. In addition to the first identified natural killer (NK) cells and lymphoid tissue inducer (LTi) cells, there are also ILC1s, ILC2s, and ILC3s (7). Moreover, ILCs derive from the common lymphoid progenitors (CLPs) and are considered to be innate counterparts of T cells (8).

The interactions between ILCs and microbiota are quite complex. On the one hand, microbiota indirectly affects ILCs through the signal transmission of accessory cells such as dendritic cells (DCs) and intestinal epithelial cells (IECs), as well as the signaling pathways of microbiota-derived metabolites and related diets; on the other hand, ILCs regulate the microbiota by producing effector cytokines such as IFN-γ produced by NK cells, IL-4 produced by ILC2s, IL-22 produced by ILC3s, as well as the participation of T and B cells. Here, we first make a brief summary of the classification, distribution, characteristic transcription factors, important effector cytokines, and functions of ILCs. Then, we focus on arranging and concluding mechanisms of the interactions between ILCs and microbiota.

### Innate lymphoid cells

#### Nomenclature and classification

NK cells were first identified and regarded as the only innate lymphoid cells (9). As more ILC subsets were discovered, they were divided into three groups based on the producing of characteristic cytokines: NK cells and ILC1s constitute Group 1 ILCs, ILC2s alone represent Group 2 ILCs and NCR^+^ILC3s, NCR^−^ILC3 and lymphoid tissue-inducer cells constitute Group 3 ILCs (10). According to similar expression of cytokines, these three groups of ILCs successively serve as innate immune counterparts for CD4^+^ T helper (Th)1, Th2, and Th17/22 cells (11). In recent years, according to the developmental trajectories and functions of the ILC subsets, ILCs have been re-divided into five subsets: NK cells, ILC1s, ILC2s, ILC3s, and LTi cells (7). In addition, several new ILC subsets were discovered. For example, regulatory ILCs (ILCregs) have an unique genetic profile and can alleviate innate intestinal inflammation (12). ILCX is independent of the three typical ILC groups, with its functions to be studied (13). A list of classification and characteristic of ILCs is presented in Table 1.

**TABLE 1:** Classification and characteristic of ILCs

Subset	Transcription factors and markers	Activation factors	Effector cytokines	Functions	Distribution
NK	T-box family T-bet EOMES NKp46, NKG2D, NK1.1, CD122	IL-2, IL-12, IL-15, IL-18	IFN-γ perforin, granzymes B	Antitumor cytotoxicity antiintracellular pathogenic microbial infection	Mainly in peripheral blood and secondary lymphoid tissues recruited to sites of inflammation upon pathogen invasion
ILC1	T-bet NKp46, NK1.1, CD122	IL-2, IL-12, IL-15, IL-18	IFN-γ	Antitumor weak cytotoxicity antiintracellular pathogenic microbial infection	Resident in mucosal tissues
ILC2	GATA3 RORα	IL-15, IL-33, TSLP	IL-4, IL-5, IL-9, IL-13 GM-CSF, Areg	Antiparasites proliferation of tuft cells and goblet cells tissue repair	Predominant in human stomach
ILC3	RORγt Ahr	IL-1β, IL-23	IL-17, IL-22 GM-CSF	Maintain mucosal barrier and homeostasis antipathogen invasion	Mainly in mucosal tissues few in lymphoid tissues
LTi	RORγt, CCR6, c-kit CXCR5, and CCR7	IL-1β, IL-23	IL-2, IL-5, IL-13, IL-17, IL-22 lymphotoxin	Maintain mucosal barrier and homeostasis antipathogen invasion formation of secondary lymph nodes and Peyer’s patch	Mainly in secondary and tertiary lymphoid structures

#### NK and ILC1s

NK cells are mainly distributed in peripheral blood, secondary lymphoid tissues, and peripheral immune organs, and are recruited to sites of inflammation upon pathogen invasion (14). ILC1s were first identified in human tonsil and ileum, and mainly resident in human mucosal tissues (15). Both NK cells’ and ILC1s’ development and functions depend on the expression of T-box transcription factor T-bet (encoded by Tbx21), with IFN-γ as the main effector cytokine (7, 13). NK cells mostly express high level of EOMES compared to lower expression in ILC1s, thus serving as a marker to distinguish between NK cells and ILC1s (16). IFN-γ, TNF-α, perforin, and granzyme produced by NK cells and ILC1s exert functions of antitumor and antiintracellular pathogenic microbial infection, as well as cytotoxic effect including induction of cell lysis and apoptosis (17 - 19). IL-2, IL-12, IL-15, and IL-18 all have different activation effect on Group 1 ILCs (16). Differently, the cytotoxic effect of ILC1s is relatively weak or even absent than that of NK cells (14).

#### ILC2s

ILC2s were originally identified in mesentery adipose and are tissue resident cells mainly found in adipose tissue, spleen, mesenteric lymph nodes, and so on (20 - 22). The characteristic transcription factor of ILC2s is GATA3. Moreover, ILC2s possess the ability to produce type 2 cytokines IL-4, IL-5, IL-6, IL-9, and IL-13, as well as amphiregulin (Areg) (7). ILC2s express receptors for IL-7 (IL-7α), IL-33 (T1/ST2), thymic stromal lymphopoietin (TSLP) in response to various cytokines and rely on Areg to repair tissues and cytokines such as IL-4 and IL-13 to mediate the activation and hyperplasia of goblet cells and tuft cells (23). Especially, ILC2s play a key role in fighting against parasites, as well as in allergic diseases such as asthma (17).

#### ILC3s

ILC3s are mainly located in mucosal tissues such as lamina propria of small intestine, with minor distribution in lymphoid tissues such as tonsil (24). ILC3s are characterized by the expression of transcription factor retinoic acid-related orphan receptor gamma subtype *t* (RORγt) and the production of cytokines such as IL-17A, IL-17F, IL-22, and granulocyte-macrophage colony-stimulating factor (GM-CSF) (25 - 27). According to the expression of NKp46 (in mouse) or NKp44 (in human) on the cell surface, ILC3s are divided into two subsets: NCR^+^ and NCR^−^ ILC3s (28). Moreover, the main homeostasis cytokine produced by ILC3s is IL-22, through which it maintains the intestinal barrier, promotes differentiation of mucus-producing goblet cells, prevents bacterial translocation, fight against the pathogen invasion, and other regulatory effect to maintain homeostasis (7). Differently, IL-17 supports the release of chemokines, which recruits proinflammatory neutrophils and may lead to intestinal inflammation (29).

#### LTi cells

LTi cells are mainly located in secondary and tertiary lymphoid structures and are critical for the formation of secondary lymph nodes and Peyer’s patch in fetal stage through lymphotoxin (30). Like ILC3s, LTi cells also strictly depend on RORγt expression, with similar expression of characteristic transcription factors and production of cytokines. Differently, LTi cells express c-Kit and CCR6, but do not express NCR (17, 19).

### Gut microbiota interact with ILCs indirectly by accessory cells

#### DCs together with macrophage and monocyte

Dendritic cells (DCs), macrophages, and monocytes first need to be activated by the microbiota, and then act as antigen-presenting cells to transmit signals to ILCs. Bacterial stimulation to DCs may be achieved by pattern recognition receptor TLR on DCs, such as TLR2 can recognize peptidoglycan and lipoteichoic acid in the cell wall of *Lactobacillus acidophilus* (*L. acidophilus*), which induces the maturation of mouse DCs and the production of IL-12 (14). Specific to NK cells, *Lactobacillus pentosus* (*L. pentossus*) strain S-PT84 can induce IFN-γ production by activating CD11c^+^DCs in TLR2 and/or TLR4 dependent manner (31). Although NK cells themselves also express TLR, bacteria cannot directly stimulate IFN-γ production by them (16, 32). It is different in term of virus that DCs produce IFN-Ⅰ (type Ⅰ interferon) stimulated by *Mouse Cytomegalo Virus* (MCMV) and *Lymphocytic Choriomeningitis Virus* (LCMV) infection, and then DCs are upregulated to trans-present IL-15 to NK cells after receiving IFN-Ⅰ stimulation through own IFN-receptors (Fig. 1) (33, 34). Among many effector cytokines produced by NK cells, it has been well studied that IL-2 can activate and enhance NK cell functions (35). Moreover, IL-15 forms a complex with IFN-Ⅰ-dependent IL-15Rα on DCs to activate NK cells in a trans-presenting manner by cell-cell contact (36). It has also been proved that IL-12 (p70), IL-18, and IL-1β can help NK cells’ activation and functions, which produced by CD11c^+^ myeloid dendritic cells (CD11c^+^mDCs) in *Salmonella typhimurium* (*S. typhimurium*) infection model *in vitro* (Fig. 1) (16). Notably, a research indicates that IL-12 is redundant in human response to mostly microorganisms (37).

**Figure:**
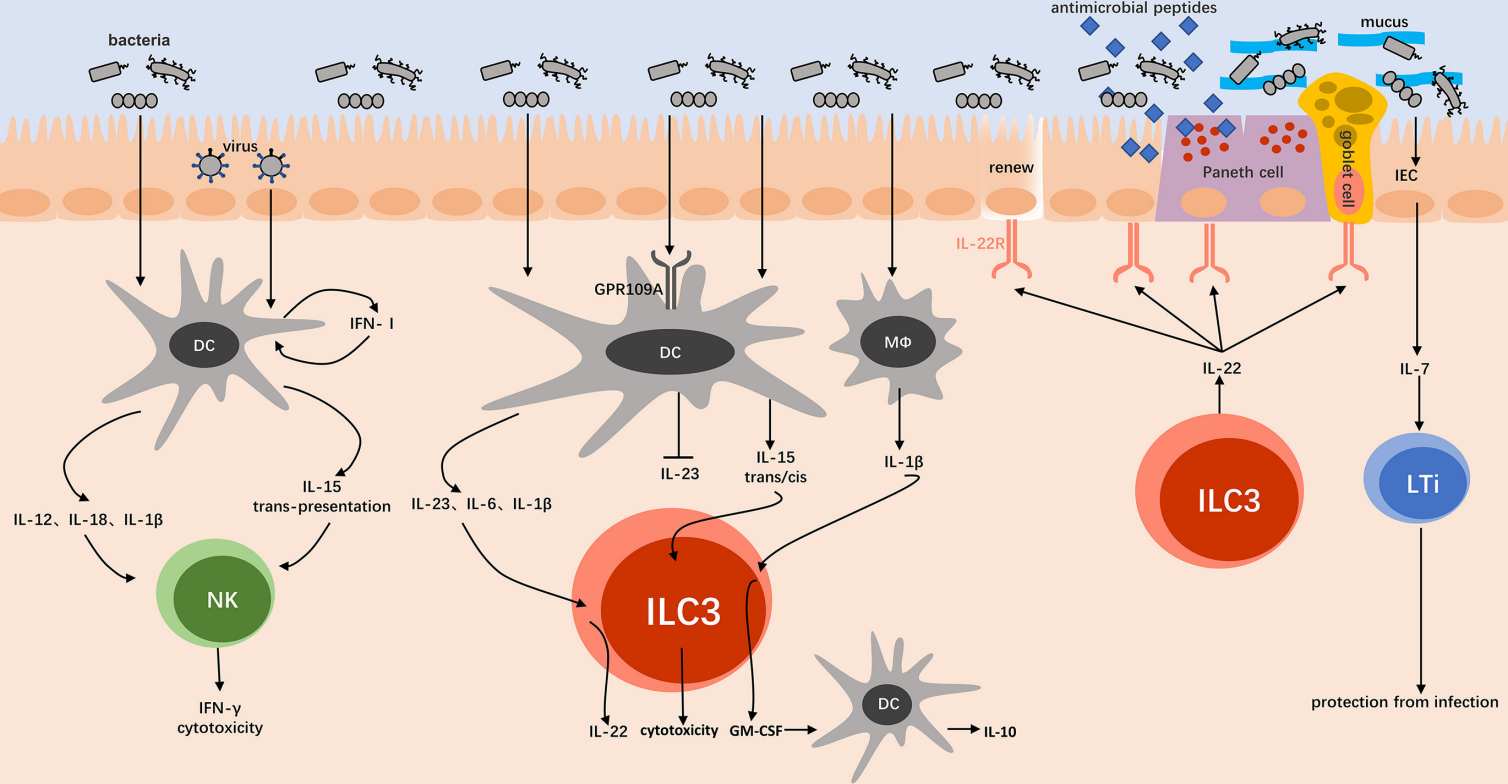
**FIG 1** Gut microbiota interact with ILCs indirectly by accessory cells. The bacteria induce the production of IL-12, IL-18, and IL-1β from DCs, which promote NK cells to secrete IFN-γ and express cytotoxicity. The viruses induce the production of IFN-Ⅰ from DCs, which promotes trans-presentation of IL-15 to NK cells, with similar consequence above. The microbiota induces the production of IL-23, IL-6, and IL-1β from DCs, which promote ILC3s to secret IL-22. The microbiota inhibits the production of IL-23 from DCs through GPR109A, which relieves inflammation in IBD. The microbiota induces the production of IL-15 from DCs, which promotes ILC3s to express cytotoxicity by both trans- and cis-presentation. GM-CSF is produced by ILC3s and can stimulate the release of IL-10 from DCs, of which process depends on IL-1β released by macrophages following microbiota stimulation. IL-22 produced by ILC3s binds to IECs through its receptor, with functions of proliferation of IECs; secretion of antimicrobial peptides; mucus-secretion of goblet cells. The microbiota induces the production of IL-7 from IECs, which promotes LTi cells to fight against infections.

In addition to NK cells, Group 3 ILCs have also been proved to require the help of DCs, macrophages, monocytes to establish connections with microbiota. *Citrobacter rodentium* (*C. rodentium*) can promote IL-22 production by ILC3s through stimulating CD11c^+^mDCs to produce IL-23 and IL-1β (Fig. 1). Notably, IL-23 and IL-1β are redundant in stimulating IL-22 production by colonic ILC3s (38). Moreover, the critical role of adaptor protein MyD88 on DCs in TLR-mediated innate immune activation through recognition of microbe-associated molecular patterns (MAMPs) has been demonstrated. *S. typhimurium* selectively enhances IL-22 production of ILC3 by secreting flagellin to activate TLR5-MyD88-IL-23 signaling pathway in antigen presenting cells (APCs), such as DCs (39). Similarly, in *C. rodentium* infection, MyD88 signaling pathway in DCs is sufficient to stimulate the expression of pro-inflammatory cytokines including IL-6, IL-1β, IL-23, and then induce the early response of Group 3 ILCs (Fig. 1) (40). GM-CSF can stimulate the release of IL-10 and other cytokines from DCs and monocytes to promote regulatory T cell (Treg) differentiation, thus maintaining intestinal tolerance. At steady state, GM-CSF is mainly produced by RORγt^+^ILC3s, and this process depends on IL-1β released by macrophages following microbiota stimulation (Fig. 1) (41). In addition to the positive regulatory pathways described above, negative ones have also been reported. In inflammatory bowel disease (IBD), GPR109a signaling pathway may inhibit the microbial-induced production of multiple inflammatory cytokines by DCs, including IL-23 in colon, thereby inhibiting ILC3s to suppress inflammation (Fig. 1) (29). Interestingly, ILC3s sometimes exhibit the characteristics of Group 1 ILCs. After bacterial stimulation, colonic mDCs express high level of IL-15Rα and trans-present IL-15 to ILC3s. Moreover, cis-presentation of IL-15 to ILC3s is also allowed because of the co-expression of all three IL-15R subunits (IL15Rα/β/γ). Ultimately, granzyme B expression of ILC3s is increased, with some ILC3 subsets even co-expressing perforin to exert a cytotoxic effect similar to NK cells (Fig. 1) (42).

#### Intestinal epithelial cells

IECs directly contact with microbiota in the gut, playing an integral role in mediating microbiota-ILCs interactions. Previous studies have focused on ILC3-dependent-IL-22 induced by microbiota (Fig. 1). IL-22 exerts functions by binding to IECs through its receptor. Mechanistically, IL-22 phosphorylates transcription factor STAT3 and modulate gene expression, after binding to its receptor (26, 43). Specifically, IL-22 promotes differentiation of mucus-producing goblet cells and maintains differentiation of crypt stem cells into IECs, which serve to protect the intestinal barrier (44, 45). Moreover, upon stimulation with IL-22, IECs and special Paneth cells produce high level of antibacterial peptides including S100A8/A9 and RegⅢγ to suppress pathogenic bacteria and maintain intestinal homeostasis (46). Besides being passively regulated by IL-22, several studies in recent years have identified that IECs can take the initiative. Depending on IFN-γ-producing NK1.1^+^ cells and IL-12, IECs can produce IL-7 induced by *C. rodentium* infection. IL-7 plays an important protective role during the early phase of infection and acts through its receptor on APCs and CD4^+^ LTi cells (Fig. 1) (47). In the same infection of *C. rodentium*, IECs’ intrinsic expression of IKKα is shown to be critical for IL-22 production by Group 3 ILCs in the gut (48).

### Microbiota interact with ILCs by microbiota-derived metabolite or related diet

#### Ahr

The aryl hydrocarbon receptor (Ahr) is an environmental sensor that works in both innate and adaptive immune systems (49). Ligands for Ahr can be derived from diet (e.g., cruciferous plants), microbial flora (e.g., *Lactobacillus tryptophan* metabolite indole), and/or host cells (50, 51). Both bacteria-derived and diet-derived ligands contribute to the maintenance of RORγt^+^ ILCs (50). It has been shown that certain bacteria in the gut can produce metabolites from tryptophan that activate Ahr *in vitro* (52). Notably, ligands derived from host cells may make a significant contribution to the development of RORγt^+^ ILCs, because deprivation of Ahr ligands from mouse diet has little effect in the accumulation of RORγt^+^ ILCs in the gut (53). Studies have demonstrated that the co-expression of Ahr and RORγt in RORγt^+^ ILCs can significantly enhance the recruitment of Ahr to the IL-22, synergistically promoting IL-22 expression and contributing to the maintenance and functions of RORγt^+^ ILCs, which play important roles in resistance to *C. rodentium* infection (Fig. 2) (54). Similarly, absent of Ahr has been proved to cause the loss of IL-22^+^ ILC3s, resulting in the decline of resistance to *C. rodentium* infection (55, 56). In addition, in a cell-intrinsic manner, Ahr signaling pathway suppresses the expression of IL-33 receptor ST2 on ILC2s mediated by Gfi1 transcription factor, and the expression of ILC2 effector molecules IL-5, IL-13, and amphiregulin. Depletion of Ahr enhances protective immunity in the gut, for instance antihelminth as well as resistence to adult *Heligmosomoides polygyrus bakeri* (*H. polygyrus bakeri*) infection (Fig. 2) (57). However, Ahr signaling pathway can also contribute to type 2-associated gut inflammation, such as ulcerative colitis and food allergy (58, 59).

**Figure:**
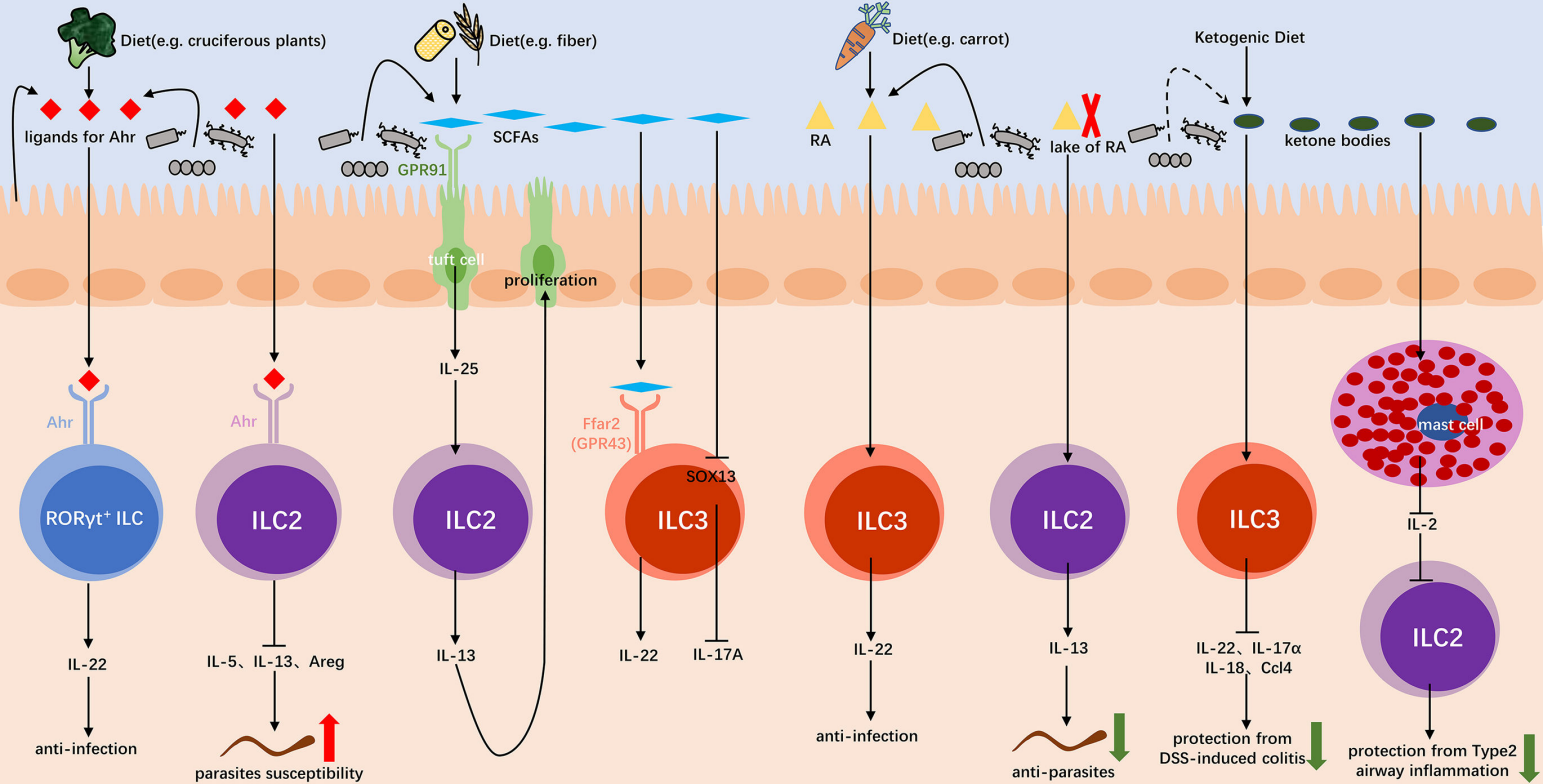
**FIG 2** Microbiota interact with ILCs by microbiota-derived metabolite or related diet The Ahr axis can promote RORγt^+^ ILCs to produce IL-22 to resist infection, and inhibit ILC2s to secret IL-5, IL-13, and Areg to be susceptible to helminth. The SCFAs axis recognized by GPR91 on tuft cells and mediated by IL-25 can promote tuft cell proliferation through IL-13 produced by ILC2s. SCFA receptor Ffar2 (GPR43) mediates the production of IL-22 by ILC3s. SCFAs reduce SOX13 expression, thus decrease IL-17A production by ILC3s. RA induce the production of IL-22 from ILC3s, which promotes antiinfection in the gut. Under a vitamin A deficient environment, increased IL-13-producing ILC2s result in enhanced resistance to nematode infections. KD can reduce ILC3s and the production of related inflammatory cytokines including IL-17α, IL-18, IL-22, and Ccl4 in DSS-induced colitis. KD can solo reduce IL-2 production by mast cells, and then reduce the proliferation of ILC2s and type 2 cytokine responses, resulting in protection from type 2 airway inflammation.

#### SCFAs

SCFAs can be produced by bacterial fermentation of dietary fiber in the colon (60). The intestinal pathosymbiont *Trichomonas* can degrade diet fiber into SCFAs, and succinate therein can be recognized by GPR91 in tuft cells. Then tuft cells secrete IL-25 to activate ILC2s, and IL-13 produced by ILC2s can in turn promote proliferation of tuft cells. It is important that the loop above can help the host fight against parasitic infections (Fig. 2) (61). Moreover, Group 3 ILCs can also sense environmental signals through SCFA receptor Ffar2 (GPR43). In murine, not only *C. rodentium* infection but also DSS-induced inflammation, Ffar2 agonists or propionate differentially activate AKT or ERK signaling pathways, and then increase ILC3-derived IL-22 through the AKT and STAT3 axis to confer protection against infection (Fig. 2) (62). In mice with hepatocellular carcinoma (HCC), gut microbiota-derived SCFAs, especially acetate, reduce SOX13 expression by inhibiting the activity of histone deacetylases. Thereby, it decreases IL-17A production by hepatic ILC3s, inhibiting tumor growth and improving prognosis (Fig. 2) (63).

#### Retinoic acid

Retonic acid (RA), a metabolite of vitamin A, is an important dietary and nutritional signal in humans. RA signaling pathway prevents and protects against colitis induced by DSS or *C. rodentium* infection through promoting IL-22 production by ILC3s to form an early immune response (Fig. 2) (64). Mechanistically, it may be that RARs on ILC3s directly bind to the promoter of IL-22, thereby regulating IL-22 mRNA transcription (64). Moreover, intestinal ILC3s express retroelement hypermethylated in cancer 1 (HIC1), in an all-trans retinoic acid (ATRA) dependent manner. It is suggested that HIC1 is required for the maintenance of IL-22-producing ILC3s in *C. rodentium* infection (65). Under a vitamin A deficient environment, severely reduced IL-22-producing ILC3s lead to impaired immunity to acute bacterial infections. However, the dramatic expansion of IL-13-producing ILC2s result in increased resistance to nematode infections (Fig. 2) (66). In addition to diet-derived RA, gut commensal bacteria such as segmented filamentous bacteria (SFB) can produce RA in the gut by expressing aldehyde dehydrogenase enzymes. This bacteria-derived RA can initiate innate immunity possibly mediated by ILCs, and promote early protection against enteric *C. rodentium* infection (67).

#### Ketogenic diet

Ketogenic diet (KD) first appeared to simulate fasting and is metabolically characterized by a low carbohydrate ratio and a high fat ratio (68). Recently, it has been shown that KD can reduce RORγt^+^CD3^−^ Group 3 ILCs and the production of related inflammatory cytokines including IL-17α, IL-18, IL-22, and Ccl4 in DSS induced colitis, protecting intestinal barrier function (Fig. 2). Notably, it has been demonstrated by fecal transplant that KD can mediate the regulation of ILC3s and the protection from colitis, depending on the modification of gut microbiota (69). Moreover, in ILC2-driven type 2 airway inflammation induced by *Alternaria alternata* (*A. alternata*), β-hydroxybutyrate (BHB) reduces the proliferation of ILC2s, type 2 cytokine responses, and immunopathology by suppressing IL-2-producing mast cells. BHB inhibits mast cell functions partly through activation of GPR109A, with similar effect found in KD and 1,3-butanediol (Fig. 2) (70).

### Microbiota interact with ILCs by adaptive immune cells participation

#### B cells secreting IgA

Although ILC2s have not been shown to directly targeted regulate commensals, previous studies have demonstrated that ILC2s can regulate the composition of microbiota indirectly by influencing IgA secretion by B cells (20, 71). In mouse model of *Helicobacter pylori* (*H. pylori*) infection, *H. pylori* enhances the induction of IL-7 and IL-33 production in the stomach, and then triggers the activation and proliferation of ILC2s. IL-5 producted by ILC2s leads to the differentiation of B cells into IgA-secreting plasma cells, and then IgA envelops the pathogen *H. pylori* and plays a role in resistence to infection (Fig. 3) (72). Similarly, in the contrast of SPF and GF mice, it can be found that commensal bacteria such as *Bacteroidaceae* family S24-7 can active ILC2s by increasing induction of gastric IL-7 production, and then upregulate IgA secretion by B cells. This process is important for gastric immune maturation, and prevents oral pathogen infection to maintain gastric homeostasis (73). Once loss the induction of commensals, such as vancomycin-mediated eradication of *Actinobacteria* and *Bacteroidetes*, it can result in a significant reduction of IgA, demonstrating that commensals are indispensable for the maintenance of gastric homeostasis (74). Notably, the upregulation of B cells secreting IgA by ILC2s is independent on the engagement of T cells, either in gastric *H. pylori* infection or in commensals (72, 73).

**Figure:**
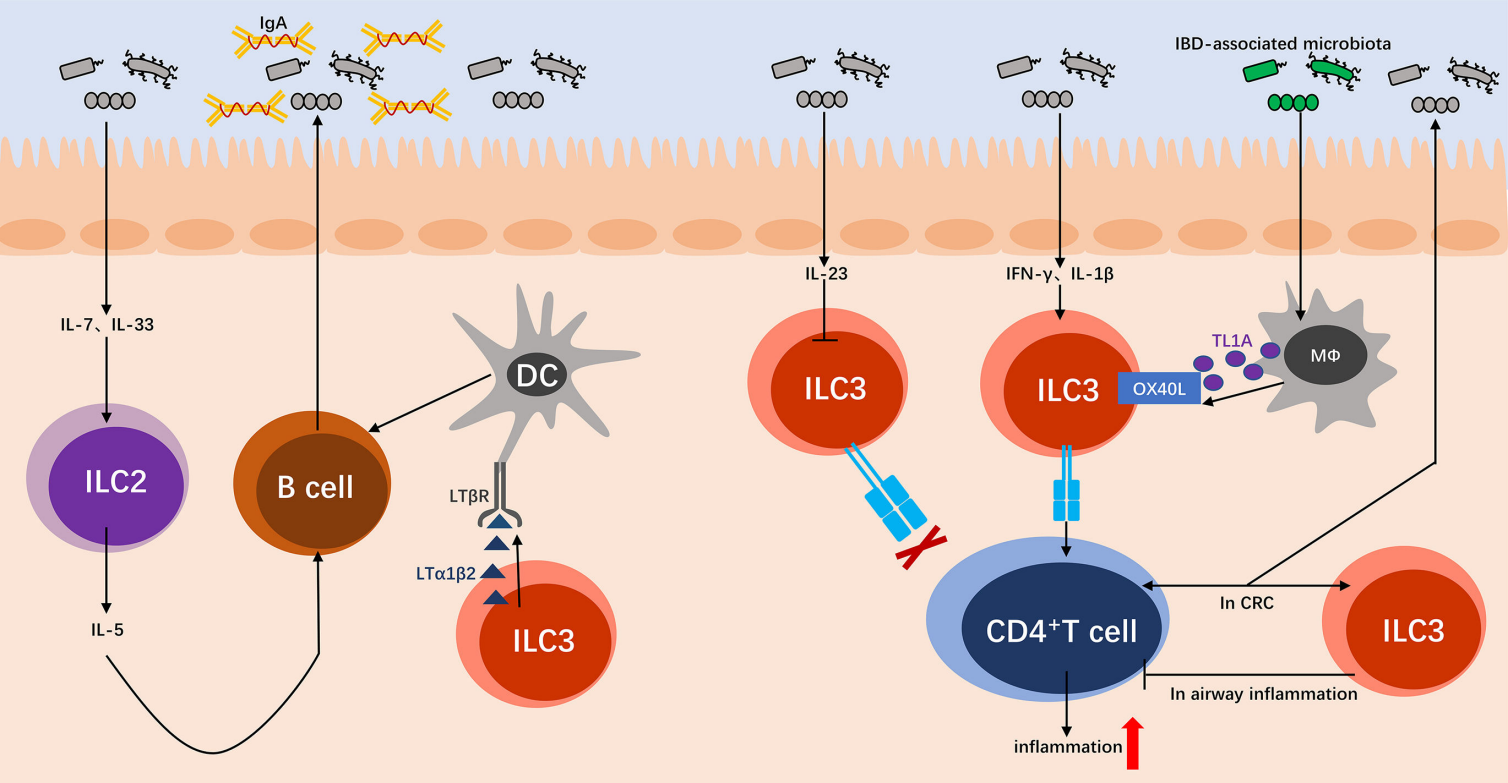
**FIG 3** Microbiota interact with ILCs by adaptive immune cells participation. The microbiota induces the production of IL-7 and IL-33, which promote ILC2s to produce IL-5, resulting in IgA secretion by B cells. In SED, B cells interact with LTβR-expression DCs which maintained by LTα1β2 from ILC3s, and then B cells class switch to IgA. The microbiota induces the production of IL-23, which inhibits MHCII expression in ILC3s. IFN-γ and IL-1β can induce CD4^+^ T cells by increased MHCII expression on ILC3s, resulting in inflammation. IBD-associated microbiota induces the release of TL1A from mononuclear phagocytes, and then enhance the expression of TL1A-dependent OX40L in MHCII^+^ ILC3s, resulting in inflammation. In colorectal cancer (CRC), the interplay between ILC3s and T cells supports the colonization of microbiota. In airway inflammation, ILC3s limit expansion of allergen-specific CD4^+^ T cells.

Moreover, accumulating evidence implicates that Group 3 ILCs are key regulators of B cells secreting IgA in the gut (46). The secretion of IgA enhances physical separation of commensals from intestinal barrier, controls the colonization balance of commensals in gut microenvironment, neutralizes potentially harmful bacterial toxins and dietary metabolites (75). In Peyer’s patches, tissue-resident B cells are activated by microbiota-derived antigens, and then upregulate CCR6 and attracted to sup-epithelial dome (SED) by CCL20. In SED, B cells interact extensively with CD11b^+^ DCs and class switch to IgA. Importantly, CD11b^+^ DCs expressing LTβR is maintained by local supply of LTα1β2 from ILC3s (Fig. 3). Unlike ILC2s, this process depends on T cells (76).

#### CD4^+^ T cells

RORγt^+^ ILCs express major histocompatibility complex class Ⅱ (MHCⅡ) and can process and present antigens. Under steady state, ILC3s restrict commensals’ specific CD4^+^ T cell pathological responses in a MHCII-dependent manner to maintain intestinal homeostasis (77). Microbiota-induced IL-23, depending on phosphorylation of mTORC1 and STAT3 in NCR^−^ ILC3s, reversibly inhibits key signals of MHCII in ILC3s. As a result, the ability that ILC3s present antigens to intestinal mucosal T cells is reduced, associating with immune tolerance (Fig. 3) (78). Moreover, MHCII^+^ ILC3s can directly induce apoptosis of activated commensal bacteria-specific CD4^+^ T cells (79, 80). However, it is different in spleen that IFN-γ can induce CD4^+^ T cells by MHCII expression on NCR^−^ ILC3s (78). Under the stimulation of IL-1β, peripheral ILC3s upregulate surface MHCII and express costimulatory molecules (Fig. 3) (81). In IBD, IBD-associated microbiota induce the release of TL1A from CX3CR1^+^ mononuclear phagocytes (MNPs), and then enhance the expression of TL1A dependent costimulatory molecule OX40L in MHCII^+^ ILC3s. This process leads to antigen-specific T cell proliferation and pathogenic Th1 cell expansion in a model of chronic colitis (Fig. 3) (82). In a model of *Helicobacter hepaticus* infection, ILC3s located in mesenteric lymph nodes are able to screen and promote the differentiation of gut microbiota-specific RORγt^+^ Tregs, and suppress Th17 cells. Thereby, it plays a critical role in establishing immune tolerance to the gut microbiota (83). In colorectal cancer (CRC) mouse model, the interplay between ILC3s and T cells through MHCII supports the colonization of microbiota, which subsequently induces type 1 immunity. Once lacking ILC3-specific MHCII, mice developed invasive CRC and resistance to anti-PD-1 immunotherapy (Fig. 3) (84). In a mouse model of house-dust-mite-induced allergic airway inflammation, antigen-presenting MHCII^+^ ILC3s significantly limit expansion of allergen-specific CD4^+^ T cells and mite-associated microbes induced Th17 cells (Fig. 3) (85).

### Conclusion and perspectives

It has been extensively studied that the role and regulatory mechanisms of ILCs in homeostasis, IBD, and cancer (18, 86, 87). However, when it comes to ILCs-microbiota interactions, few articles have made systematic summary. In this review, we cite the latest research advances to dissect the mechanisms of ILCs-microbiota interactions as far as possible.

In the first major mechanism of accessory cells, DCs are considered as the key role because most of the interactions between ILCs and microbiota rely on mediators secreted by DCs. In addition to DCs, macrophages and monocytes of both APCs and IECs of non-APCs are also involved. Moreover, some studies have found that glial cells can regulate ILC3s production of IL-22 in an MYD88-dependent manner to protect the gut against pathogenic bacteria (88, 89). Of course, it remains to be investigated that the role of glial cells and other accessory cells in the interaction of ILCs with microbiota.

In the second major mechanism of metabolic pathways, it is worth thinking about whether dietary therapy can be used as an effective therapeutic manner to improve the interactions between ILCs and microbiota. In addition to KD which playing a protection and mitigation role in mouse IBD, it is worthwhile to investigate whether other metabolic pathways can affect bacterial susceptibility or disease progression through changes in diet.

In the third major mechanism of adaptive immune cells, not only IgA-secreting B cells can directly provide immune effect, but also MHCII-presented CD4^+^ T cells can provide immune tolerance. Furthermore, the latest study found that the ratio of Treg to Th17 in the intervening CD4^+^ T cells was closely related to ILCs and microbiota (83). Therefore, the selective role of ILCs on gut microbiota-specific Tregs and other CD4^+^ T cell subsets may be focus of the following study.

Finally, in addition to the three major mechanisms of ILC-microbiota interactions above, it remains to be explored and refined. We look forward to establishing a complete ILC-microbiota interaction framework that will contribute to human health.
